# Prospective plasma lipid profiling in individuals with and without depression

**DOI:** 10.1186/s12944-018-0796-3

**Published:** 2018-06-26

**Authors:** Dietmar Enko, Wolfgang Brandmayr, Gabriele Halwachs-Baumann, Wolfgang J. Schnedl, Andreas Meinitzer, Gernot Kriegshäuser

**Affiliations:** 1 Institute of Clinical Chemistry and Laboratory Medicine, General Hospital Steyr, Sierningerstraße 170, 4400 Steyr, Austria; 2 Clinical Institute of Medical and Chemical Laboratory Diagnostics, Medical University of Graz, Auenbruggerplatz 15, 8036 Graz, Austria; 3 Department of Psychiatry and Psychotherapeutic Medicine, General Hospital Steyr, Sierningerstraße 170, 4400 Steyr, Austria; 4 Practice for General Internal Medicine, Dr. Theodor-Körner-Straße 19b, 8600 Bruck/Mur, Austria

**Keywords:** Biomarkers, Cholesterol, Depression, Lipids, Triglycerides

## Abstract

**Background:**

So far, studies on possible association of plasma lipid levels and depressive disorder are contradictory. This prospective work aimed at assessing a plasma lipid profile in individuals with major depression and healthy controls.

**Methods:**

In total, 94 patients with major depression and 152 healthy controls were included in this prospective study. After an overnight fasting state of 12 h they underwent blood drawing for triglyzerides (TG), total cholesterol, low-density lipoprotein (LDL)- and high-density lipoprotein (HDL)-cholesterol measurements. All participants were evaluated in a clinical interview and filled out the self-rating Beck Depression Inventory (BDI-II) scale to identify depressive symptomatology.

**Results:**

Ninety-four patients with major depression showed significantly higher median (interquartile range) plasma TG levels (108.0 [75.8–154.1] vs. 84.0 [63.0–132.2] mg/dL, *P* = 0.014) and significantly lower HDL-cholesterol levels (55.0 [46.9–123.0] vs. 61.5 [47.4–72.6] mg/dL, *P* = 0.049) compared to 152 individuals without depression, respectively. Total and LDL-cholesterol concentrations were observed slightly higher in patients with major depression. Significant positive correlation was found between TG, total cholesterol and LDL-cholesterol concentrations and the BDI-II score (*p* = 0.027, 0.048 and 0.018), and in tendency negative correlation between HDL-cholesterol levels and the BDI-II score (*P* = 0.091), respectively.

**Conclusions:**

Depressive individuals were found with adverse plasma lipid patterns of higher TG and lower HDL-cholesterol levels compared to healthy controls. On this basis, the authors would suggest the implementation of routine lipid measurements in order to stratify these patients by their cardiovascular risk.

## Background

In the last years the interest in the relationship between plasma lipid composition and disease increased rapidly. In clinical routine, total cholesterol, low-density lipoprotein (LDL)- and high-density lipoprotein (HDL)-cholesterol, and triglycerides (TG) are used as basic lipid parameters to identify individuals at risk for cardiovascular disease.

Hyperlipidemia (i.e., hypercholesterinemia and/or hypertriglyceridemia) is a well-established risk factor for cardiovascular disease [1]. In addition, depression must be considered as an independent risk factor for myocardial infarction and increased mortality in patients with coronary heart disease [2, 3]. However, well-documented predictive laboratory biomarkers of depression are still lacking, and the question raises, if depression might be associated with an altered plasma lipid pattern.

In the current literature, findings regarding a possible link between depression and blood lipid levels are contradictory. A number of previous studies have found an association between lower total cholesterol and depression [4–7]. Other reports indicate, that depression might be related to higher blood cholesterol levels [8–10]. Inconsistent findings were also obtained with the less extensively assessed LDL- and HDL-cholesterol, and TG [6, 7, 11].

These mixed results reflect the complexity of possible associations between depressive disorders and lipid biomarkers. Discrepant findings, however, may be explained by a broad spectrum of different study designs and methodological variations (i.e., restricted age range, small sample size, heterogenous study populations) [11, 12]. For this reason the comparability of studies and the generalizability of outcomes is limited [11].

In addition, previous reports not always considered TG levels in their study design [5, 10, 12]. This type of major circulating lipid is responsible for maintaining the body’s energy balance and might also be involved in the development of cognitive impairment in individuals suffering from a depressive disorder [13].

To address the above mentioned limitations of previous reports, the present work was performed in a carefully evaluated homogenous psychiatric cohort with depression out of any other psychiatric comorbidities. This study aimed at investigating possible alterations of TG, total cholesterol, and LDL- and HDL-cholesterol plasma levels in a large sample of well-characterized patients including men and women over a broad age range sub-grouped by the presence or absence of major depression.

## Methods

### Participants and study design

A total of 246 participants aged between 18 and 70 years were recruited for this prospective study. The baseline characteristics of the study population are shown in Table 1. The average age was 40.5 ± 14.8 years. Eighty-eight (35.77%) individuals were male and 158 (64.23%) were female.

**Table 1:** Baseline characteristics of all individuals included in this study (*n* = 246)

	Minimum	1st quartile	Median	3rd quartile	Maximum
Age (years)	18	27	39	52	70
Height (m)	1.53	1.65	1.70	1.78	1.98
Weight (kg)	20	60	71	83	142
BMI (kg/m^2^)	7.35	21.47	24.14	27.73	49.13
Triglycerides (mg/dL)	28	65	97	138	312
Total cholesterol (mg/dL)	112	167	191	219.3	320
LDL cholesterol (mg/dL)	50	94	116	145	228
HDL cholesterol (mg/dL)	33	47	58	70.1	123
BDI-II score	0	3	10	20	50

Abbreviations: *BMI* body mass index, *LDL* low-density lipoprotein, *HDL* high-density lipoprotein, *BDI-II* Beck depression inventory

Out of these individuals, 94 suffered from a major depressive disorder without any other psychiatric comorbidity. They were diagnosed and treated by experienced psychiatrists at the Department of Psychiatry and Psychotherapeutic Medicine (General Hospital Steyr, Steyr, Austria). A total of 152 individuals with neither a depressive symptomatology nor a former history of psychiatric disorder, who visited the outpatient clinic of the Institute of Clinical Chemistry and Laboratory Medicine (General Hospital Steyr, Steyr, Austria) for a medical health check-up served as healthy controls. The gender and age groups of individuals with and without depression are presented in Table 2.

**Table 2:** Gender and age groups of individuals with and without depression

	Depression			Non depression		
	Female (n)	Male (n)	Total (n)	Female (n)	Male (n)	Total (n)
Age groups (years)						
18–99	24	10	34	32	10	42
30–49	20	9	29	37	29	66
50–70	20	11	31	25	19	44
Total	64	30	94	94	58	152

All study participants filled out the Beck Depression Inventory (BDI-II) questionnaire [14] and were investigated for the complete lipid status (i.e., TG, total cholesterol, LDL- and HDL-cholesterol). At study entry, none of the patients were under medication of lipid-lowering agents. Height (cm) and weight (kg) were measured with a wall-mounted metric tape and a calibrated personal scale to calculate the body mass index (BMI) (kg/m^2^).

### Blood collection and clinical laboratory procedures

Blood samples were drawn into VACUETTE® LH lithium heparin tubes (4 mL) (Greiner Bio-one International GmbH, Kremsmünster, Austria) after an overnight fasting state (12 h) in the morning between 08.00 and 10.00. Samples were then centrifuged at 2000 x g for 10 min. All analyses were performed within one day after blood collection. Plasma TG (desirable level ≤ 150 mg/dL), total cholesterol (desirable level ≤ 200 mg/dL), LDL-cholesterol (desirable level depends on cardiovascular risk) and HDL-cholesterol (desirable level > 40 mg/dL) were measured using commercial enzymatic methodology on a Dimension Vista® 1500 System (Siemens Healthcare GmbH, Vienna, Austria).

### BDI-II

The 21-question multiple-choice survey (scale: 0–3) was used to measure the severity of depression. The total values of BDI-II range between 0 and 63 points [15].

### Statistical analysis

Descriptive statistics were performed to summarize and present the study parameters. The distribution of data was calculated with the Kolmogorov-Smirnov test. As the observed metric values were not normally distributed, the exact Mann-Whitney U-test was used for subgroup comparisons. Not normally distributed data were described as medians (Q1 – Q3). A *P*-value of < 0.05 was considered statistically significant. For all calculations, the Analyse-it® software version 4.92 (Analyse-it Software, Ltd., Leeds, United Kingdom) was used.

## Results

### Lipid measurements

**Fig. 1:**
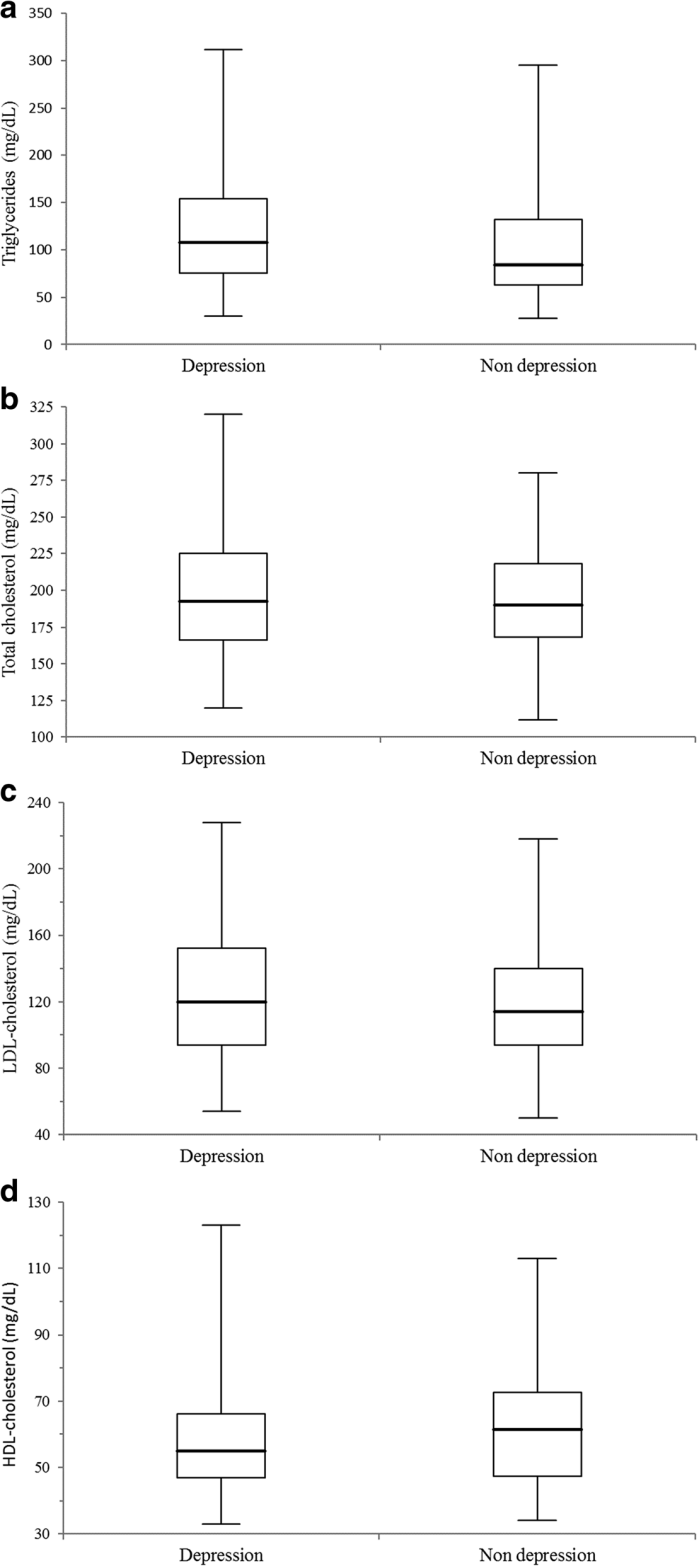
Box-and-whisker plots of **a** TG, **b** total cholesterol, **c** LDL-cholesterol, and **d** HDL-cholesterol plasma level comparisons between 94 patients with major depression and 152 healthy controls (*P*-values were 0.014, 0.529, 0.269, and 0.049). The central boxes represent the 25th to 75th percentile range. The lines inside the boxes show the median value for each group

All in all, 53 (21.15%) out of 246 subjects investigated here had plasma TG levels > 150 mg/dL, and 104 (42.3%) total cholesterol concentrations > 200 mg/dL, respectively. The plasma lipid patterns obtained for all individual investigated here are shown in Table 3. Patients with major depressive disorder (*n* = 94) had significantly higher median (interquartile range) plasma TG levels (108.0 [75.8–154.1] mg/dL) compared to healthy controls (*n* = 152) (84.0 [63.0–132.2] mg/dL) (*P* = 0.014). Moreover, HDL-cholesterol was significantly lowered (55.0 [46.9–123.0] mg/dL) in subjects with major depression compared to the healthy control group (61.5 [47.4–72.6] mg/dL) (*P* = 0.049). Total and LDL-cholesterol concentrations were observed slightly higher in patients with major depression. The box-and-whisker plots of the complete lipid status are illustrated in Fig. 1 (a-d).

**Table 3:** Study parameters observed for 94 patients with major depression and 152 healthy controls

	Depressive patients	Healthy controls	*P*-value
Height (m)	1.68 (1.65–1.78)	1.70 (1.64–1.77)	0.931
Weight (kg)	74.5 (60.0–90.0)	70.0 (60.0–80.6)	0.214
BMI (kg/m^2^)	25.11 (21.36–28.50)	23.95 (21.53–26.43)	0.134
Triglycerides (mg/dL)	108.0 (75.8–154.1)	84.0 (63.0–132.2)	0.014
Total cholesterol (mg/dL)	192.5 (166.0–225.2)	190.0 (168.0–218.0)	0.529
LDL cholesterol (mg/dL)	120.0 (93.8–152.0)	114.0 (94.0–140.0)	0.269
HDL cholesterol (mg/dL)	55.0 (46.9–123.0)	61.5 (47.4–72.6)	0.049
BD-II score	25 (18–33)	5 (2–9)	< 0.001

Abbreviations: *BMI* body mass index, *LDL* low-density lipoprotein, *HDL* high-density lipoprotein, *BDI-II* Beck depression inventory

Data are presented as medians (Q1 – Q3)

Subgroup analysis of males and females and different age groups showed higher TG, total and LDL-cholesterol and lower HDL cholesterol plasma concentrations in patients with major depression compared to healthy controls in all groups. In females, individuals with major depression (*n* = 64) had significantly higher plasma TG (98.5 [66.8–133.5] mg/dL vs. 80.0 [61.8–121.0] mg/dL, *P* = 0.022) and significantly lower HDL-cholesterol levels (59.5 [49.0–70.6] mg/dL vs. 66.5 [55.8–76.1] mg/dL, *P* = 0.036) compared to healthy controls (*n* = 94), whereas differences of plasma TG (117.5 [97.8–161.2] mg/dL vs. 104.5 [67.9–154.6] mg/dL, *P* = 0.243) and HDL-cholesterol concentrations (47.0 [42.9–54.2] mg/dL vs. 49.0 [42.0–64.1] mg/dL, *P* = 0.317) were not significant between depressive (*n* = 30) and non-depressive males (*n* = 58), respectively (Table 2).

### Correlations between lipid parameters and severity of depressive symptoms

There was a positive correlation between BDI-II score and TG (*P* = 0.027, Pearson’s correlation coefficient 0.141), total cholesterol (*P* = 0.048, Pearson’s correlation coefficient 0.126), and LDL-cholesterol (*P* = 0.018, Pearson’s correlation coefficient 0.150), and in tendency a negative correlation with HDL-cholesterol (*P* = 0.091, Pearson’s correlation coefficient − 0.108), respectively. HDL-cholesterol showed a highly inverse correlation with TG (*P* < 0.001, Pearson’s correlation coefficient − 0.456).

## Discussion

In the present study, 94 patients with major depressive disorder and 152 healthy controls were assessed and compared for TG, total cholesterol, LDL- and HDL-cholesterol. Individuals with depression were found to have significantly higher plasma TG (108.0 [75.8–154.1] vs. 84.0 [63.0 vs. 132.2] mg/dL, *P* = 0.014) and lowered HDL-cholesterol levels (55.0 [46.9–123.0] vs. 61.5 [47.4 vs. 72.6] mg/dL, *P* = 0.049) compared to healthy subjects. Total and LDL-cholesterol measurements were observed slightly higher in individuals with depression. These findings are in agreement with two recently published studies that found higher levels of TG, total and LDL-cholesterol and lower HDL-cholesterol levels in individuals with depressive disorder versus healthy controls [8, 16].

In contrast, a previous study by Aijänseppä et al. reported concordant TG concentrations and lower total and LDL-cholesterol measurements in subjects with depression as compared to individuals without depression [17]. One recently published work reported higher TG and also higher HDL-cholesterol levels in depressive individuals [18]. Study design variations and heterogeneity of study participants are possible explanations for these inconsistent findings. While the present study investigated 246 individuals (female: 158) with a mean age of 40.5 years, Aijänseppä et al. studied 421 men with a mean age of 76.8 years. A clear advantage of our study was the approach of a carefully evaluated homogenous psychiatric cohort with depression out of any other psychiatric comorbidities.

Herein, individuals suffering from major depression were observed with significantly elevated TG levels which also positively correlated with disease severity. One previous Finnish longitudinal study, determining serum lipid level patterns (i.e., TG, LDL- and HDL-cholesterol) and depressive symptoms during childhood and early adulthood, indicated, that rapid increases of TG at early age were predictive of depression onset [19]. However, the investigators failed to demonstrate an association between any LDL- or HDL-cholesterol pattern change and depressive symptoms [19].

Adverse lipid patterns in depressive disorder are associated with life-style related factors. Individuals with depression were reported to eat more often foods high in energy density compared to healthy controls [20]. This cofactor might be one possible reason for positive associations between plasma lipid levels and depression found in this study.

Recently, elevated TG levels and depression severity were shown to play a major role in the development of cardio-metabolic disease [21]. Depression is known to be associated with an increased activity of the sympathetic nervous system and the hypothalamic-pituitary-adrenal cortex axis [20, 22]. Cortisol increases serum levels of circulating free fatty acids [20, 23], which in turn stimulate the very-low density lipoprotein (VLDL) synthesis in the liver resulting in elevated TG concentrations [20].

Present data showed, that in subjects with major depression significantly elevated TG levels coincided with significantly lowered HDL-cholesterol in a highly inverse correlated manner (*P* < 0.001, Pearson’s correlation coefficient − 0.456). A previous study found this adverse lipid pattern associated with an increased risk of recurrent or chronic depression [24]. Moreover, recent evidence suggests that elevated TG levels associated with lowered HDL-cholesterol predispose to atherosclerosis [24–26]. Raised TG are indicative of high remnant cholesterol concentrations, leading to intimal low-grade inflammation and accumulation of foam cells and atherosclerotic plaques [25]. Regarding the findings of this study, TG and HDL-cholesterol measurements may have the potential to serve as screening biomarkers in individuals with depression thereby contributing to the preservation of long-term cardiovascular health.

The major limitation of this cross-sectional study is that cortisol measurements and lifestyle-related factors such as dietary habits, alcohol use or smoking behaviour were not taken into account. Furthermore, we cannot rule out the influence of antidepressant medication on the patients´ lipid metabolism. However, prospective longitudinal studies including follow-up measurements of blood lipid levels are warranted to fully elucidate the associations with depressive disorders.

## Conclusions

Individuals with depressive disorder showed higher TG, total and LDL-cholesterol and lower HDL-cholesterol plasma levels compared to healthy controls. On this basis, the authors would suggest the implementation of routine blood lipid measurements in order to stratify these patients by their cardiovascular risk.

## Abbreviations

BDI-II: Beck Depression Inventory
BMI: body mass index
HDL: high-density lipoprotein
LDL: low-density lipoprotein
TG: triglycerides
VLDL: very-low density lipoprotein
